# PRDM Proteins Orchestrate Colorectal Cancer Tumorigenesis

**DOI:** 10.3390/ijms27083392

**Published:** 2026-04-09

**Authors:** Erika Di Zazzo, Carmela Sorrentino, Monica Rienzo, Donatella Fiore, Maria Chiara Proto, Amelia Casamassimi, Patrizia Gazzerro, Ciro Abbondanza

**Affiliations:** 1 Department of Medicine and Health Sciences “V. Tiberio”, University of Molise, 86100 Campobasso, Italy; erika.dizazzo@unimol.it; 2 Department of Precision Medicine, University of Campania “Luigi Vanvitelli”, 80138 Naples, Italy; carmela.sorrentino@unicampania.it (C.S.); amelia.casamassimi@unicampania.it (A.C.); ciro.abbondanza@unicampania.it (C.A.); 3 Department of Environmental, Biological, and Pharmaceutical Sciences and Technologies, University of Campania “Luigi Vanvitelli”, 81100 Caserta, Italy; 4 Department of Pharmacy, University of Salerno, 84084 Fisciano, Italy; dfiore@unisa.it (D.F.); pgazzerro@unisa.it (P.G.); 5 Department of Medicine and Surgery, University of Enna “Kore”, 94100 Enna, Italy; mariachiara.proto@unikore.it

**Keywords:** colorectal cancer, PRDM, cancer biomarkers, oncogene, tumor suppressor, consensus molecular subtypes (CMS)

## Abstract

Colorectal cancer (CRC) is a heterogeneous disease driven by complex genetic, epigenetic, and microenvironmental alterations. Members of the PR domain-containing (PRDM) protein family have emerged as context-dependent regulators of CRC initiation, progression, tumor cell plasticity, immune modulation, and therapeutic response. Accumulating evidence highlights divergent roles for PRDM proteins as tumor suppressors, oncogenes, or isoform-dependent dual-function regulators. Collectively, PRDM family members represent a central node of transcriptional/epigenetic control in CRC biology, with significant potential as biomarkers for early detection, prognosis, and treatment stratification, as well as promising candidates for epigenetic and pathway-directed therapeutic strategies.

## 1. Introduction

**Figure 1:**
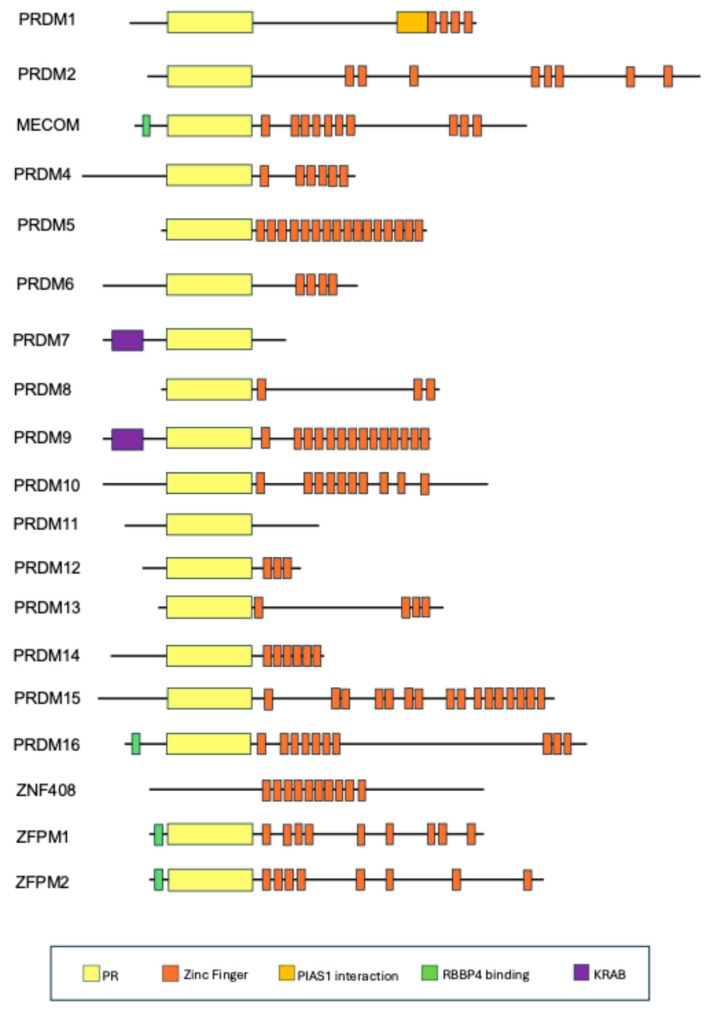
Domain organization of human PRDM proteins. PR domains are shown in yellow and C2H2 zinc finger motifs in orange. Additional functional domains are indicated, including KRAB domains (purple), RBBP4 binding domains (green), and PIAS1 interaction regions. The schematic representation highlights the structural diversity among PRDM family members in terms of domain composition and organization.

The *PRDM* gene family, also named PRDI-BF1 (positive regulatory domain I-binding factor 1) and RIZ1 (retinoblastoma protein-interacting zinc finger gene 1), encodes a group of transcriptional regulators belonging to the Kruppel-like zinc finger superfamily [1,2]. PRDM proteins share a conserved PR (PRDI-BF1–RIZ1 homologous) domain and C-terminal zinc finger motifs, which mediate DNA binding, protein–protein interactions, and nuclear localization [3]. In humans, 17 to 19 PRDM family members have been identified [3]. Indeed, after the discovery of a previously unrecognized PR domain in their encoding genes, two further members have been recently added to this family: zinc finger protein, FOG (friend of GATA-1) family member 1 (*ZFPM1*; FOG1) and zinc finger protein, FOG family member 2 (*ZFPM2*; FOG2) [2,3,4,5]. The PR domain is evolutionarily related to the SET [Su(var)3-9, enhancer-of-zeste, trithorax] domain characteristic of histone lysine methyltransferases; however, intrinsic catalytic activity has been demonstrated only for a limited subset of PRDM proteins (Figure 1) [1,2,3,4].

The tumor-suppressive activity of the PR domain relies on both catalytic and non-catalytic mechanisms that converge on chromatin structure regulation and transcriptional repression. Structurally related to SET domains, the PR domain in some PRDM proteins (e.g., PRDM2, PRDM7, PRDM9) exhibits intrinsic histone methyltransferase (HMT) activity, primarily targeting lysine residues such as H3K9, a modification associated with transcriptional repression [1,4].

In many PRDM family members lacking catalytic activity, the PR domain instead functions as a scaffold that recruits chromatin-modifying complexes, including histone deacetylases (HDACs), lysine demethylases (e.g., LSD1/KDM1A), and protein arginine methyltransferases (e.g., PRMT5), thereby regulating transcriptional programs associated with gene repression [1,2,3].

Through these interactions, PR domain-containing isoforms promote the establishment of transcriptionally repressive chromatin states at promoters and enhancers of oncogenes, thereby limiting proliferation, stemness, and survival pathways [2,4].

Rather than acting primarily as enzymes, most PRDM proteins regulate chromatin through protein–protein interactions within transcriptional and epigenetic multiprotein complexes. By directly or indirectly associating with chromatin-modifying enzymes, including HMTs, PRMT5, LSD1/KDM1A, histone deacetylases, and histone acetyltransferases, PRDMs modulate chromatin structure and gene expression [1,3]. Depending on the cellular and molecular context, PRDM proteins can either bind specific DNA sequences through their zinc finger domains or act as transcriptional cofactors without direct DNA contact. Through these mechanisms, PRDM proteins contribute to stem cell maintenance, cell differentiation, metabolic regulation, and signaling pathways that control cell fate and tissue homeostasis [2,3]. Of note, both direct methyltransferase activity (documented for a few family members such as PRDM2, PRDM7 and PRDM9) and indirect recruitment of epigenetic “writers” and “erasers” have been described, highlighting the dual enzymatic and scaffold roles of PRDM proteins [4].

**Figure 2:**
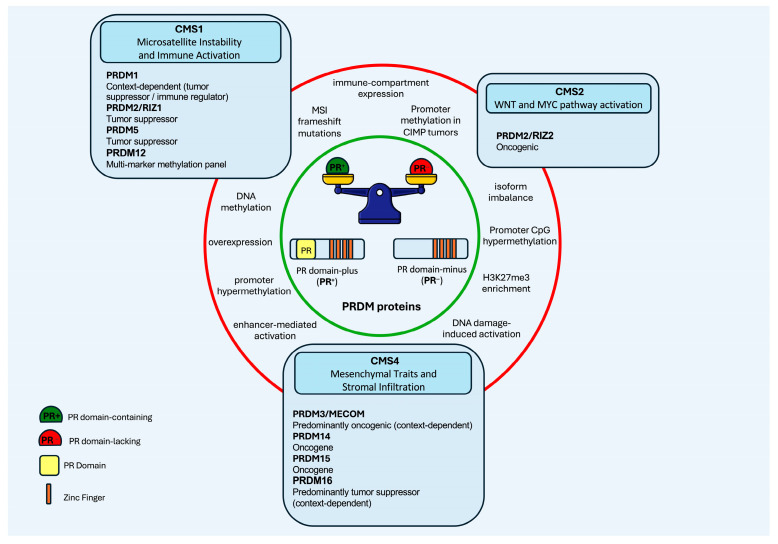
Schematic overview of PRDM family members in CRC. The central panel illustrates the balance between PR domain-containing (PR^+^) and PR domain-lacking (PR^−^) isoforms, which can be generated through alternative promoter usage and/or alternative splicing. PR^+^ isoforms are mainly tumor suppressive, whereas PR^−^ isoforms are associated with oncogenic functions. The green circle indicates a physiological equilibrium between PR^+^ and PR^−^ isoforms, while the red circle highlights a shift toward PR unbalance, associated with oncogenic activation and altered tumor behavior. Bidirectional relationships reflect context-dependent roles or isoform switching (e.g., PRDM2/RIZ1 vs. PRDM2/RIZ2). The figure summarizes regulatory mechanisms, including promoter hypermethylation, MSI-associated mutations, enhancer-mediated activation, histone modifications (e.g., H3K27me3), and isoform imbalance, which collectively shape PRDM expression and CRC subtype behavior.

A recurrent feature of *PRDM* genes is the expression of distinct protein isoforms that differ in the presence or absence of the PR domain. Full-length PR-positive (PR^+^) isoforms and truncated PR-negative (PR^−^) variants arise through alternative promoter usage or splicing events and often exert divergent biological effects (Figure 2). In cancer, PR^+^ isoforms are commonly associated with growth-restrictive and tumor-suppressive functions, whereas PR^−^ variants tend to promote proliferation and cell survival. Disruption of the PR^+^/PR^−^ balance, through genetic alterations, epigenetic silencing, or selective transcriptional activation, has been reported in multiple tumor types and is now recognized as a recurrent mechanism contributing to malignant progression [1,2,3]. This “Yin–Yang” model represents a central paradigm in PRDM biology and explains their isoform-specific oncogenic or tumor-suppressive outcomes [4].

Colorectal carcinoma (CRC) represents a major global health burden and is characterized by marked inter- and intra-tumoral heterogeneity at both genetic and epigenetic levels [6]. Despite improvements in early diagnosis and multimodal treatment strategies, including surgery, chemotherapy and targeted therapies against VEGF/VEGFR and EGFR, advanced CRC remains associated with high relapse rates and limited long-term survival. Tumor recurrence has been partially attributed to therapy-resistant cancer stem cell (CSC) populations, which survive to conventional antiproliferative treatments and sustain tumor growth [7]. These CSC-driven processes are supported by key signaling pathways, including Wnt/β-catenin, EGFR, IGF-1R, and NOTCH, that regulate the balance between proliferation and differentiation in CRC [6,7].

To better capture this biological complexity, the consensus molecular subtype (CMS) classification stratifies CRC into four major subgroups based on transcriptomic and molecular features [8]. CMS1 tumors are characterized by microsatellite instability (MSI) and immune activation; CMS2 by canonical WNT and MYC pathway activation; CMS3 by metabolic dysregulation; and CMS4 by mesenchymal traits and stromal infiltration. Although CMS classification has improved prognostic stratification, its predictive value for therapeutic response remains debated [9], highlighting the need to identify additional molecular determinants contributing to CRC heterogeneity and treatment resistance.

Given their central role in chromatin regulation and pathway integration, PRDM proteins are well positioned to influence CMS-associated molecular programs. Different PRDM members intersect with signaling networks that differ among CMS groups, including WNT/MYC signaling, immune-related programs, metabolic reprogramming, and growth factor signaling. Interpreting PRDM alterations within the CMS framework may therefore clarify their context-dependent functions in CRC. Indeed, several PRDM family members undergo genetic mutations, transcriptional deregulation, or epigenetic modifications in CRC, leading to altered chromatin organization and perturbation of key oncogenic pathways [3,10]. Among these, PRDM2 represents one of the best-characterized examples. *PRDM2* is frequently affected by frameshift mutations, particularly in MSI tumors, and by promoter hypermethylation in premalignant serrated lesions. These alterations result in an imbalance between its PR^+^ (PRDM2/RIZ1) and PR^−^ (PRDM2/RIZ2) isoforms and correlate with epigenetic reprogramming and tumor progression [11,12,13]. Functionally, the PR^−^ isoform PRDM2/RIZ2 has been shown to engage the EGF/EGFR signaling axis in CRC models, linking extracellular growth factor stimulation to transcriptional responses and potentially influencing sensitivity to EGFR-targeted therapies [14]. Beyond *PRDM2*, *PRDM5* has been consistently reported as epigenetically silenced in a subset of CRC tumors, where loss of *PRDM5* is associated with enhanced Wnt signaling and increased proliferative capacity, supporting a tumor-suppressive role for this PRDM family member [3,15].

Despite accumulating evidence implicates PRDM proteins in CRC, many aspects of their biology remain unresolved. In particular, the contribution of less extensively studied family members (e.g., PRDM3/MECOM, PRDM9, PRDM10, PRDM16), the functional relevance of isoform diversity across the family, and the integration of PRDM-mediated epigenetic regulation with signaling heterogeneity and tumor cell plasticity, including cancer stem cell biology, require further investigation [4,10]. Addressing these questions will be essential to fully define the role of PRDMs in CRC carcinogenesis and to evaluate their potential as biomarkers or therapeutic targets.

Recent evidence indicates that CMS labels of CRC capture not only tumor-intrinsic transcriptional programs but also varying degrees of stromal and immune admixture, and that different regions within the same tumor can map to distinct CMS-like states. Spatial transcriptomics has revealed pronounced intra-tumoral CMS heterogeneity and neighbourhood-specific programs that are not detectable by bulk classification, with mesenchymal/stromal-rich niches driving CMS4-like signals adjacent to epithelial regions resembling CMS2/3 [16]. Consistently, spatial proteomics further supports CMS-dependent organization of cancer–stroma–immune interactions, highlighting that prognostic and therapy-relevant signals can be compartmentalized rather than uniform across the tumor mass [16,17]. These data have two direct implications for PRDM biology in CRC: (i) PRDM expression and function should ideally be interpreted in a CMS-stratified manner, while also accounting for spatial context and cell-type specificity; and (ii) apparent “oncogenic” or “tumor-suppressive” effects may reflect differential abundance of PRDM-expressing compartments (epithelium vs. stromal/immune) rather than a single uniform tumor-cell state [18].

In this review, we synthesize current evidence on PRDM proteins in CRC, focusing on their molecular architecture, epigenetic functions, genetic and transcriptional alterations, isoform-specific activities, and their relevance within CMS stratification (Table 1 and Figure 2).

## 2. PRDM Proteins Involved in CRC

### 2.1. PRDM1

PRDM1, also known as BLIMP1 (B lymphocyte-induced maturation protein-1), was first identified as a repressor of human β-interferon gene (*IFNB1*) expression. It is a key regulator of embryonic development and terminal differentiation in numerous cell lineages, including immune cells. It is a well-established tumor suppressor gene in human DLBCL (diffuse large B cell lymphoma) and in other hematological malignancies [3].

The *PRDM1* gene has two alternative promoters, transcribing two isoforms (PRDM1α and PRDM1β) with opposite functions; they are both still able to bind to DNA although only the first one includes the PR domain [2,3,4].

PRDM1 also exerts context-dependent functions in CRC, influencing tumor cell proliferation, stemness, stress adaptation, and immune regulation.

A retrospective study, comparing clinicopathological, molecular, and prognostic factors between left-sided (LCC) and right-sided (RCC) CRC, showed that *PRDM1* mutations correlated with shorter progression-free survival in RCC [40]. Multiple independent studies support a tumor-suppressive role for PRDM1 in CRC. Activation of PRDM1 by genkwadaphnin (GD-1), a bioactive compound derived from Daphne genkwa, markedly inhibited proliferation of the metastatic CRC cell line SW620 [19]. GD-1 activated PKD1 and MEK signaling, which increased *PRDM1* expression, suppressed MYC, and induced the cyclin-dependent kinase inhibitor CDKN1A resulting in cell-cycle arrest and reduced CRC cell growth. PKD1 silencing abrogated PRDM1 induction and reversed the effects on MYC and CDKN1A, confirming a PKD1–PRDM1 signaling axis [19].

Consistent with this tumor-suppressive function, transcriptomic analyses of a cohort of 217 CRC cases revealed a significant downregulation of *PRDM1* in tumor tissues [41]. Moreover, *PRDM1β* has been identified as a direct TP53-responsive gene [42]. A link between PRDM1 and TP53-mediated response had been already observed through previous siRNA studies in human CRC cells [43]. *PRDM1* silencing resulted in both apoptosis and growth arrest. In these cells, both *TP53* mRNA and protein levels increased together with the induction of TP53-target genes. These effects were largely abrogated in cells lacking or depleted in *TP53*. Furthermore, PRDM1 was able to bind *TP53* promoter and repress its transcription thus suggesting an autoregulatory feedback loop [43]. A study using *TP53* codon 72 (Pro72Arg, rs1042522) variants in RKO CRC cells demonstrated allele-specific transcriptional programs following DNA damage, with *PRDM1β* emerging as an Arg72-specific TP53 target [42]. Functional assays showed that PRDM1 isoforms repress MYC-driven transcriptional networks and stemness-associated genes. In human colon organoids, forced *PRDM1* expression suppressed tumor organoid formation and growth, whereas reduced *PRDM1* expression correlated with poorer survival outcomes in CRC patients [20]. Collectively, these findings indicate that TP53-mediated tumor suppression is partly executed through *PRDM1*-dependent epigenetic silencing of stem cell programs, thereby limiting malignant expansion following genotoxic stress.

A further molecular mechanism in CRC was uncovered using microarray and luciferase assay; indeed, miR-223-3p promoted CRC development at least partly through repression of *PRDM1* expression [44].

In contrast to its growth-inhibitory effects under certain conditions, PRDM1 may also contribute to tumor adaptation in sporadic CRC. Ribosomal dysfunction triggered by cellular stress induces transcriptional reprogramming, and PRDM1 has been identified as a key mediator of this response in both CRC patient samples and murine models. Stress-induced ribosomal impairment elevates *PRDM1* levels in intestinal cancer cells, promoting survival and enhancing cancer stemness during treatment exposure. Mechanistically, PRDM1 facilitates clustering and modulation of insulin-like growth factor (*IGF*) receptor-associated genes, thereby supporting tumor progenitor survival and disease progression [45]. These findings suggest that PRDM1 may have context-dependent functions, acting either as a tumor suppressor or as a mediator of stress adaptation.

Beyond tumor-intrinsic mechanisms, PRDM1 shapes the immune microenvironment in CRC. Elevated *PRDM1* expression has been observed in CRC tumor tissues and proposed as a potential marker of T-cell exhaustion with prognostic relevance [21]. *PRDM1*–expressing regulatory T cells (Tregs) within tumors display a more activated phenotype compared with conventional Tregs and may influence antitumor immunity [46,47].

PRDM1-dependent programs associated with T-cell exhaustion and regulatory T-cell activation [21,46,47] are consistent with the immune-dominant microenvironment characteristic of CMS1 tumors [8]. Nevertheless, direct CMS-stratified analyses of *PRDM1* expression and function remain limited.

These observations underscore a dual role for PRDM1 in CRC, encompassing both direct regulation of cancer cell behavior and modulation of immune responses within the tumor niche.

PRDM1 biology is particularly relevant to immune-rich CRC microenvironment, where exhausted or terminally differentiated T-cell states and activated regulatory programs shape antitumor immunity. A recent study reinforces the centrality of PRDM1/BLIMP1 in enforcing terminal differentiation trajectories that are mechanistically coupled to exhaustion-related transcriptional programs [48]. In parallel, *PRDM1* can also be expressed in malignant compartments with immunosuppressive consequences, underscoring the need to distinguish immune versus tumor-cell *PRDM1* signals when interpreting bulk CRC datasets [49]. These aspects align conceptually with CMS1 (MSI–immune) and potentially with immune-infiltrated CMS4 contexts, but specific CMS- and spatially resolved analyses remain necessary to determine whether PRDM1-driven programs are primarily involved in immune-microenvironment, tumor-intrinsic, or both [16].

Taken together, evidence from multiple experimental systems, including cell lines, organoids, animal models, and patient cohorts, supports a central role for PRDM1 in CRC as both a tumor-intrinsic regulator and a key modulator of the tumor immune microenvironment.

### 2.2. PRDM2

PRDM2 (also known as RIZ) was first identified in independent studies as retinoblastoma interacting zinc-finger protein (RIZ) and as GATA3 binding protein through functional screenings of human cDNA libraries. Since then, further variants have been isolated, including PR-less isoforms containing only the C-terminal zinc finger domains. As mentioned above, the imbalance in the RIZ1/RIZ2 amounts may represent an important cause of several malignancies [2,3,4,50].

Genetic and epigenetic alterations of *PRDM2* have been consistently linked to CRC risk, onset and progression. *PRDM2* maps to chromosome 1p36, a locus frequently deleted in multiple malignancies, further supporting its tumor-suppressive role [50,51,52]. In addition, an association of a *PRDM2* polymorphic allele with some cancers, including CRC, was detected [53]. Of note, a significant association between CRC susceptibility and a functional *PRDM2* single-nucleotide polymorphism (S450N) was reported in postmenopausal women [54]. More recently, the *PRDM2* promoter hypermethylation, leading to the selective repression of the *PRDM2/RIZ1* isoform, has been identified as a potential early event in the progression of sessile serrated lesions toward dysplasia and CRC [13].

Multiple independent studies have demonstrated that *PRDM2/RIZ1* is preferentially mutated or silenced in MSI-positive tumors [55]. *PRDM2/RIZ* mutations were detected in approximately 31% of MSI CRCs but were absent in microsatellite-stable (MSS) tumors, with significant associations observed with female sex, proximal tumor location, early stage, and poor differentiation [56]. Frameshift mutations of *RIZ* are particularly prevalent in hereditary nonpolyposis CRC across all disease stages [57,58]. Particularly, frameshift mutations affecting polyadenine tracts—(A)8 or (A)9—within exon 8 of *PRDM2* occur in approximately one third of MSI-positive CRCs, often resulting in reduced or absent *RIZ1* mRNA and truncated protein products [59,60]. Restoration of *RIZ1* expression in MSI CRC cell lines induced G2/M cell-cycle arrest and apoptosis and suppressed tumor growth in xenograft models, further supporting its tumor-suppressive function [61].

Functional evidence for the oncogenic relevance of these alterations was provided by genome editing studies in HCT116 cells, in which correction of the *PRDM2* c.4467delA mutation restored normal function. Re-expression of *PRDM2* led to increased global H3K9 dimethylation and significantly reduced cell migration, anchorage-independent growth, and tumorigenicity in vivo. Transcriptomic analyses further identified regulation of multiple cancer-related pathways, notably epithelial–mesenchymal transition (EMT), with vimentin (VIM) emerging as the most significantly affected gene. These data support *PRDM2* c.4467delA as a functionally relevant driver alteration in CRC [22].

In silico analyses of The Cancer Genome Atlas (TCGA) datasets confirmed that *PRDM2* is frequently mutated and transcriptionally deregulated in CRC [10]. Notably, increased expression of the *PRDM2* isoform *PRDM2/RIZ2* strongly correlated with downregulation of the tumor-suppressive isoform *PRDM2/RIZ1*. RNA-sequencing data revealed that *PRDM2/RIZ2* overexpression profoundly reshapes the CRC transcriptome through deregulation of EGF signaling, implicating RIZ2 in the autocrine EGF-mediated regulation of DLD1 cell behavior. Consistently, forced *PRDM2/RIZ2* expression enhanced cell viability, proliferation, colony formation, migration, and organoid growth, effects likely driven by elevated EGF secretion [14].

The direct link of the PR^−^ isoform (PRDM2/RIZ2) to transcriptional rewiring centered on EGF/EGFR signaling, supports a mechanistic interface with CMS2 “canonical” biology where WNT/MYC and growth factor-linked proliferative programs predominate [14]. This provides a testable framework to evaluate RIZ1/RIZ2 imbalance as a subtype-specific determinant of sensitivity to EGFR-targeted strategies, ideally in CMS-stratified cohorts and organoid models [18].

Beyond tumorigenesis, *PRDM2* has also emerged as a potential predictive biomarker of response to chemoradiotherapy in patients with locally advanced rectal cancer [62]. Furthermore, MSI-associated mutations in *PRDM2*, together with alterations in TGFβ-RII and IGF-IIR, have been linked to enhanced cellular proliferation and increased sensitivity to 5-fluorouracil in CRC cell lines [63]. As CMS1 tumors are typically MSI-positive [8], the preferential occurrence of *PRDM2* alterations in MSI-positive CRC suggests potential relevance in CMS1-associated disease. Taken together, these findings support a preferential association of *PRDM2* alterations with MSI-high tumors, which predominantly correspond to CMS1. In parallel, the involvement of *PRDM2* (particularly the RIZ2 isoform) in EGF/EGFR signaling suggests a functional overlap with CMS2 tumors, which are characterized by epithelial differentiation and activation of growth factor-driven proliferative programs [8]. However, CMS-stratified analyses of *PRDM2* mutation and isoform imbalance remain limited [8,14].

Collectively, genetic, epigenetic, and functional evidence supports a central role for PRDM2 in CRC pathogenesis, highlighting its potential as both a biomarker and a therapeutic target.

### 2.3. MECOM

*PRDM3/MECOM* (MDS1 and EVI1 Complex) locus was first identified as a site of proviral insertion in murine myeloid leukemias. This locus is formed by the fusion of two coding genes with two distinct transcription starting sites and isoform subgroups produced by alternative splicing events, myelodysplasia syndrome 1 (MDS1) and ecotropic virus integration site 1 (EVI1). Hence, the PR^+^ product is formed by combining the two genes whereas the PR^−^ isoform, named EVI1 or sPRDM3 (short PRDM3), is transcribed separately and is a potent oncogene in several leukemia subtypes [2,3,4].

Although *MECOM* was originally characterized as an oncogene in hematologic malignancies, subsequent studies have shown that its function in many solid tumors, including CRC, is highly context-dependent. In CRC, both tumor-promoting and tumor-suppressive activities have been described, reflecting the biological complexity of this transcription factor. Elevated *MECOM* expression has been consistently associated with advanced disease and unfavorable outcomes in CRC. Increased *MECOM/EVI1* levels correlate with higher tumor stage, lymph node involvement, distant metastasis, and poor differentiation [64].

Early genetic studies identified *MECOM* as a target of frameshift mutations in MSI–high (MSI-H) CRC. The gene contains an A7 mononucleotide repeat that is particularly susceptible to replication errors in MSI-H cancers. These mutations were detected exclusively in MSI-H CRCs and exhibited intratumoral heterogeneity, highlighting the genetic diversity within this tumor subtype [65]. In addition, *MECOM* has been reported among candidate genes located within recurrently gained genomic regions, further supporting its involvement in CRC tumorigenesis.

Beyond genetic alterations, epigenetic mechanisms involving *MECOM* have been described. Tumor-specific super-enhancers (SEs) have been recognized as key regulators of oncogene activation in CRC. A SE located upstream of the *ETS2* oncogene was identified in both inflammatory bowel disease (IBD) and CRC tissues. This regulatory element physically interacts with the *ETS2* promoter and is required for its transcriptional activation. Notably, enhancer activity and enhancer RNA transcription were markedly increased in IBD and CRC compared with normal colon tissue, correlating with elevated *ETS2* expression. An IBD-associated risk single nucleotide polymorphism within this SE was shown to alter MECOM binding, thereby influencing *ETS2* transcription. Functional experiments demonstrated that MECOM cooperates with ETS2 to sustain CRC cell proliferation, clonogenic growth, sphere formation, and migration, linking non-coding genetic variation, epigenetic regulation, and oncogenic signaling [24].

At the mechanistic level, MECOM/EVI1 promotes metastatic dissemination through epigenetic repression of metastasis suppressor genes. It directly binds to the promoter of *TIMP2* and recruits DNMT1, leading to DNA methylation and transcriptional silencing. Pharmacological inhibition of DNA methylation restored *TIMP2* expression and significantly reduced metastatic burden in vivo, underscoring the therapeutic relevance of targeting epigenetic modifications in *EVI1*-overexpressing tumors [23]. MECOM/EVI1 also exerts complex effects on EMT. Although it represses the EMT regulator SLUG and its depletion induces EMT-like morphological changes in CRC cells, MECOM/EVI1 remains necessary for efficient metastatic colonization in vivo. These findings indicate that EMT induction and metastatic competence may be partially uncoupled in CRC, with MECOM/EVI1 primarily enhancing metastatic fitness rather than acting as a classical EMT driver [66].

Additional regulatory functions of MECOM/EVI1 include repression of ΔNp63, which results in CDKN1A-mediated cell-cycle arrest in TP53-deficient CRC cells [67], and suppression of miR-143, a negative regulator of KRAS signaling [68]. *MECOM/EVI1* overexpression is also observed in CRC and adenomas, where it enables tumor cells to evade TGFβ-mediated growth inhibition through interaction with Smad proteins, thereby disrupting tumor-suppressive TGFβ signaling [69]. Moreover, MECOM/EVI1 directly regulates anti-apoptotic genes such as *BCL-xL*, promoting tumor cell survival [70].

Chronic inflammation, a recognized driver of CRC development, has also been linked to MECOM alterations. In a mutational screen of inflammation-associated colon tumors, *MECOM* emerged among recurrently mutated genes implicated in pathways regulating cellular senescence and TGFβ signaling. Functional studies in mice demonstrated that Mecom can exert tumor-suppressive effects in this inflammatory context, suggesting that disruption of senescence-related pathways contributes to inflammation-driven CRC and that MECOM may participate in this regulatory network [71].

Altered glycosylation patterns, another hallmark of cancer, have also been associated with MECOM expression in CRC. Comprehensive glycomic profiling of multiple CRC cell lines revealed distinct signatures across N-glycans, O-glycans, and glycosphingolipids. Cancer cell lines were enriched in (sialyl)Lewis antigens, whereas poorly differentiated cells showed increased expression of blood group A, B, and H antigens. Integrative analyses combining glycomic and transcriptomic data identified strong positive correlations between (sialyl)Lewis antigens, the fucosyltransferase *FUT3*, and *MECOM* expression, suggesting that MECOM may contribute to the regulation of glycosylation programs associated with tumor progression and metastatic potential [72,73].

Given its multifaceted involvement in CRC progression, therapeutic targeting of MECOM-related pathways has attracted interest. The natural compound matrine has been shown to induce apoptosis and G0/G1 cell-cycle arrest in CRC cells by upregulating miR-22, which directly targets *MECOM*. This modulation leads to suppression of Wnt/β-catenin and MEK/ERK signaling pathways, highlighting MECOM-related pathways as a potential strategy for therapeutic intervention in CRC [74].

*MECOM* has also been incorporated into multigene mRNA-based models designed to predict response to preoperative chemoradiotherapy in rectal cancer, supporting its potential utility in patient stratification [25]. Furthermore, high *MECOM* expression has been linked to resistance to irinotecan, possibly through activation of MAPK signaling pathways [75].

Because MECOM integrates epigenetic repression, TGFβ signaling, EMT-associated programs, and inflammatory signals, its functional profile is consistent with key features of CMS4 tumors. This type is characterized by mesenchymal activation, strong stromal infiltration, and TGFβ-driven transcriptional programs, all of which overlap with *MECOM*-dependent signaling pathways [8]. However, direct CMS-stratified analyses of *MECOM* expression and activity remain limited.

### 2.4. PRDM5

*PRDM5* is a well-established tumor suppressor gene in a multitude of cancer types where it is often silenced through CpG methylation. It encodes an epigenetic regulator that negatively regulates the transcription of WNT reporters and WNT-responsive genes thus modulating WNT/beta-catenin signaling [3,4].

*PRDM5* is frequently silenced also in CRC through promoter CpG island hypermethylation and trimethylation of histone H3 at lysine 27 (H3K27me3). In primary tumors, *PRDM5* methylation is detected in a subset of CRCs, while adjacent non-neoplastic tissues remain unmethylated, underscoring its cancer-specific epigenetic inactivation and potential biomarker value [27].

Subsequent analyses across cancer and precursor lesion cohorts revealed that *PRDM5* promoter methylation is significantly enriched in *BRAF*-mutant CRCs and is strongly associated with advanced disease, the CpG island methylator phenotype (CIMP), and the serrated neoplasia pathway. Serrated polyps display intermediate levels of *PRDM5* methylation, whereas conventional adenomas exhibit minimal methylation, suggesting a stepwise epigenetic silencing process. Immunohistochemical analyses demonstrate progressive loss of PRDM5 protein from early lesions to invasive cancers, regardless of *BRAF* mutation status, indicating that *PRDM5* downregulation is an early and pervasive event in colorectal tumorigenesis. Notably, *PRDM5* mutations are rare, reinforcing the predominance of epigenetic mechanisms in its inactivation [15]. The restoration of PRDM5 expression markedly suppresses CRC cell proliferation, supporting its role as a negative regulator of gastrointestinal tumorigenesis [27].

Further studies substantiated the tumor-suppressive role of PRDM5. Re-expression of PRDM5 via recombinant adenoviral delivery induces G2/M cell-cycle arrest and apoptosis in tumor cells, directly linking PRDM5 loss to malignant phenotypes [76]. In vivo, PRDM5 protein expression is reduced in human colorectal neoplastic lesions, and genetic deletion of *Prdm5* in mice significantly increases adenoma burden in the context of an Apc^Min background, providing compelling evidence for its role in restraining intestinal tumor initiation and progression [26].

The enrichment of *PRDM5* methylation in CIMP-positive and serrated lesions is consistent with epigenetically defined CRC subsets.

Beyond its established tumor suppressor role and frequent epigenetic silencing in CRC neoplasia, *PRDM5* loss should be interpreted within the broader framework of the serrated pathway and CIMP. Promoter hypermethylation of *PRDM5* has been reported as an early event in serrated lesions and advanced adenomas, suggesting that its inactivation may participate in epigenetic field defects preceding invasive carcinoma [15]. Given that CIMP-high tumors frequently overlap with *BRAF*-mutated and MSI-high CRCs, molecular features commonly enriched within the immune-associated CMS1 subtype, the possibility that *PRDM5* silencing preferentially characterizes specific molecular routes of tumorigenesis warrants further investigation. The clinical and biological relevance of consensus molecular subtypes in stratifying CRC heterogeneity has been extensively validated and provides a suitable framework for subtype-oriented analyses [8,77]. While current data support *PRDM5* as a tumor suppressor frequently inactivated through promoter methylation, its potential value as a prognostic or subtype-specific biomarker remains insufficiently defined. CMS-stratified and longitudinal studies may clarify whether *PRDM5* methylation represents merely an early epigenetic alteration or identifies tumors following a distinct evolutionary trajectory with specific clinical implications.

These findings underscore *PRDM5* silencing as being supported by converging epigenetic, functional, and in vivo evidence, and highlight its relevance as an early biomarker candidate and a promising target for epigenetic-based therapeutic strategies in CRC.

### 2.5. PRDM10

PRDM10 has recently been shown to function in early embryonic development through transcriptional activation, which is mediated by binding to highly conserved DNA motifs and recruitment of coactivators via its glutamine-rich domain, which is uniquely present at the C-terminus of PRDM10 among the family members [4].

A study using integrated bioinformatics and network analysis recognized the role of *PRDM10* as one of the important differential genes between grade II and grade III of rectum cancer [28]. Consistently, in our previous pan-cancer analysis based on TCGA datasets [9], *PRDM10* alterations were characterized by both gene mutations and increased expression levels in multiple tumor types. In that study, gene expression profiles were derived from TCGA deposited exome and RNA-Seq data and analyzed using standardized pipelines for expression profiling, allowing the identification of *PRDM10* dysregulation across cancers. As a possible mechanism, PRDM10 protein was found to affect *BCL2* gene expression at the transcription level, thus influencing apoptosis, in many cancers including CRC [29].

Although PRDM10 has been less extensively characterized in CRC compared with other PRDM family members, its involvement in transcriptional regulation of apoptosis-related genes suggests a broader role in maintaining tumor cell survival networks. Given the recurrent dysregulation of apoptotic pathways in CRC, particularly in therapy-resistant contexts, PRDM10 may contribute to tumor progression through modulation of survival signaling. However, its functional relevance in defined molecular subtypes and its potential interaction with canonical CRC pathways such as WNT or EGFR signaling remain largely unexplored.

### 2.6. PRDM12

PRDM12 is essential for the neurogenesis initiation and activation of a cascade of downstream pro-neuronal transcription factors in the nociceptive lineage. *PRDM12* mutations have been linked to congenital insensitivity to pain, since they occur in a homozygous fashion and affect the development of sensory neurons destined to differentiate into nociceptors [4].

Comprehensive profiling of CpG island methylation in CIMP-positive and CIMP-negative CRC tissues, alongside matched adjacent normal mucosa, identified *PRDM12* as a previously unrecognized epigenetic marker in CRC. *PRDM12* exhibited low baseline methylation in normal colon tissue, consistent with restricted gene expression, whereas tumor samples showed increased methylation accompanied by aberrant expression patterns. Incorporation of *PRDM12* methylation status into a multimarker diagnostic panel, together with established markers FOXE1 and SDC2, significantly enhanced the performance of methylated stool DNA (sDNA), a robust, non-invasive approach for early CRC detection [30].

The emergence of *PRDM12* methylation as part of multi-target stool DNA panels highlights the growing relevance of epigenetic biomarkers in CRC screening strategies. These findings support the additive value of PRDM12 within composite multi-marker assays and reinforce the concept that epigenetic alterations represent early and clinically exploitable events in colorectal tumorigenesis [78].

From a biological standpoint, it remains unclear whether *PRDM12* methylation preferentially associates with specific molecular routes of colorectal carcinogenesis, such as CIMP-high or MSI-driven tumors, which are frequently enriched within CMS1. The frequent methylation of *PRDM12* in CIMP-high tumors suggests a potential association with CMS1, which is characterized by widespread CpG island methylation and immune activation. This supports the inclusion of *PRDM12* within epigenetic biomarker panels relevant to this subtype, although CMS-stratified validation remains limited [8]. The molecular and clinical stratification of CRC into consensus molecular subtypes has been extensively characterized and provides a framework for such analyses [18].

Importantly, while current evidence supports the diagnostic value of *PRDM12* methylation in screening settings, data regarding its prognostic or predictive significance remain limited, underscoring the need for subtype-stratified validation in prospective cohorts.

### 2.7. PRDM14

PRDM14 plays a key role in pluripotency and stem cell maintenance, whose dysregulation has been widely implicated in cancer. Frequently overexpressed or amplified in several malignancies, including breast, lung, and pancreatic cancers, *PRDM14* has been associated with increased proliferation, metastasis, and poor clinical outcome [2,3,4]. Mechanistically, it promotes cancer stem cell-like phenotypes through epigenetic reprogramming, including modulation of DNA methylation and regulation of signaling pathways involved in cell fate and survival. However, epigenetic silencing of *PRDM14* has also been described in specific tumor contexts, indicating that its functional role may vary depending on the biological setting [2,3,4].

*PRDM14* has been identified as a consistently dysregulated gene in CRC by integration of multiple Gene Expression Omnibus (GEO) microarray datasets [79]. In addition, previous work had revealed significant *PRDM14* hypermethylation in colon carcinogenesis [80].

PRDM14 shows particularly high expression levels at the invasive tumor front, where its expression correlates with lymph node metastasis and advanced disease stage [31]. Kaplan–Meier analyses demonstrated its strong prognostic value for patient survival. In particular, multivariate analyses identified elevated PRDM14 as an independent prognostic factor in patients with stage III CRC [31,79]. Functional studies demonstrate that PRDM14 overexpression enhances invasiveness, chemoresistance, and stem-like properties in vitro, while promoting tumorigenicity in vivo, collectively implicating PRDM14 as a driver of CRC progression and therapeutic resistance [31].

Mechanistically, PRDM14 has been linked to the oncogenic Hippo pathway signaling through functional interplay with the transcriptional co-activator YAP1. Although sustained YAP1 activity is essential for proliferation and tumor maintenance in many cancers, PRDM14 can compensate for *YAP1* loss by restoring cell growth and tumorigenic potential in CRC cell lines, xenograft models, and patient-derived colon organoids. Both YAP1 and PRDM14 converge on the transcriptional activation of *CALM2* and *SLC2A1*, genes required for PRDM14-mediated rescue of *YAP1* suppression. These findings position PRDM14-dependent regulation of calcium signaling and glucose transport as a critical component of YAP1-driven oncogenesis [32].

The functional coupling between PRDM14 and YAP1-dependent oncogenic programs in CRC, validated across cell lines, xenografts and CRC organoids, supports a model in which PRDM14 can sustain growth under constraints on Hippo signaling [32]. Given the established role of YAP1 signaling in promoting mesenchymal traits and stromal interactions, PRDM14-driven activation of the Hippo/YAP1 axis may align with CMS4 features, which include enhanced invasiveness, EMT, and microenvironmental remodeling [8].

Genome-wide plasma cell-free DNA methylome analysis using MeDIP-seq revealed significant hypermethylation of *PRDM14* in CRC patients compared with healthy controls, underscoring its potential utility as a blood-based biomarker for clinical application [33]. Moreover, in silico drug-repositioning approaches have identified potential anti-neoplastic and immunomodulatory agents targeting PRDM14-associated pathways [79].

### 2.8. PRDM15

PRDM15 is a transcriptional regulator with emerging roles in development and cancer [3,4]. Initially identified as a candidate tumor suppressor due to recurrent deletions at chromosome 21q22 in pancreatic cancer cell lines, it has also been reported as overexpressed or mutated in hematological malignancies. Mechanistically, PRDM15 regulates key signaling pathways for the safeguard of naive pluripotency, including WNT and MAPK-ERK, through direct control of gene transcription. These findings suggest a context-dependent role of PRDM15 in tumorigenesis [2,3,4].

*PRDM15* is markedly upregulated in CRC, and its expression correlates with advanced disease stage and unfavorable prognosis. Mechanistic analyses identified PRDM15 as a previously unrecognized suppressor of TP53 signaling, acting through transcriptional repression of USP10 to destabilize TP53. Genetic ablation of *PRDM15* inhibits CRC cell proliferation by inducing TP53-dependent cell-cycle arrest and apoptosis, leading to significant suppression of tumor growth in vitro and in vivo. Beyond tumor growth control, PRDM15 plays a critical role in mediating therapeutic response and radio-resistance in CRC. *PRDM15* depletion enhances the sensitivity of CRC cells to 5-fluorouracil, establishing the PRDM15–USP10–TP53 axis as a key driver of CRC progression and a potential therapeutic vulnerability [35].

Moreover, following DNA damage, *PRDM15* is rapidly upregulated and recruited to sites of double-strand breaks, where it co-localizes with γ-H2AX and engages the DNA-PKcs–Ku70/Ku80 repair machinery. Loss of *PRDM15* compromises DNA repair capacity and markedly increases radiosensitivity in CRC cells. Consistently, *PRDM15* silencing significantly enhances radiotherapy efficacy in both cell-derived and patient-derived rectal cancer xenograft models [34].

Beyond its mechanistic involvement in DNA repair and TP53 destabilization, PRDM15 should be interpreted within the broader molecular taxonomy of CRC. The enrichment of therapy resistance stromal activation, and mesenchymal signaling in CMS4 tumors suggests that *PRDM15*-driven programs are functionally consistent with this subtype. CMS4 tumors are characterized by TGFβ activation, EMT, and strong microenvironmental interactions, features that overlap with *PRDM15*-mediated regulation of DNA damage response and therapeutic resistance [8]. While direct CMS-stratified analyses are currently lacking, integrating *PRDM15* expression with CMS classification in rectal cancer cohorts treated with neoadjuvant chemoradiotherapy could clarify its predictive value for treatment response [18].

Furthermore, given the growing evidence of intra-tumoral CMS heterogeneity revealed by spatial transcriptomics, it remains to be determined whether *PRDM15* expression is restricted to malignant epithelial cells or also detectable in stromal compartments contributing to therapy resistance niches. Such compartment-specific analyses may refine its potential as a biomarker and therapeutic vulnerability [16].

Collectively, these findings identify PRDM15 as a key regulator of therapeutic resistance and tumor progression, supporting its potential utility as both a predictive biomarker for radiotherapy response and a rational target for therapeutic intervention [34].

### 2.9. PRDM16

PRDM16, originally identified as MEL1 (MDS1/EV1-like gene 1), is a PRDM family member structurally related to MECOM/EVI1 that plays a crucial role in the determination and function of brown and beige fat [3,4]. In cancer, it was initially characterized in hematological malignancies, where it is frequently involved in chromosomal rearrangements and expressed as both full-length (PR^+^) and short (PR^−^) isoforms. The short isoform (sPRDM16), lacking the PR domain, has been associated with leukemogenesis through impaired differentiation and dysregulation of signaling pathways such as TGF-β. Beyond hematological cancers, PRDM16 alterations, including copy number variations, epigenetic deregulation, and aberrant expression, have been reported in several solid tumors, where it may exert context-dependent oncogenic or tumor-suppressive functions [3,4].

*PRDM16* has been identified among candidate oncogenes located in gained focal minimal common genomic regions of MSS CRC [81]. Although initially proposed as a candidate driver within recurrently gained regions, subsequent functional studies indicate that PRDM16 predominantly exerts tumor-suppressive effects in CRC.

Epigenetic regulation of *PRDM16* also appears to play an important role in CRC. An epigenome-wide DNA methylation analysis of visceral adipose tissue (VAT) from 25 healthy individuals and 29 CRC patients, performed using the Infinium HumanMethylation450K BeadChip, revealed a global increase in DNA methylation levels in CRC patients compared to controls. A distinct methylation pattern involving *PRDM16* in VAT was found to be associated with CRC [82]. This finding suggests a potential link between systemic epigenetic alterations and CRC, although tumor-specific validation in primary tumor tissue remains necessary.

Further studies have demonstrated that aberrant DNA methylation of *PRDM16* is associated with CRC metastasis. PRDM16 expression is significantly reduced in CRC tissues and is closely correlated with larger tumor size, advanced T stage, and poorer overall and disease-free survival. Functional analyses showed that *PRDM16* downregulation enhances CRC cell proliferation, migration, and invasion both in vitro and in vivo, primarily through modulation of the EMT pathway. Treatment with the DNA methylation inhibitor decitabine restores *PRDM16* expression and suppresses CRC progression. Mechanistically, PRDM16 interacts with peroxisome proliferator-activated receptor gamma (PPARG) in the nucleus and enhances its expression. PPARG levels are lower in CRC tissues compared with adjacent normal mucosa. Functionally, PPARG inhibits CRC cell proliferation, colony formation, migration, and invasion through regulation of the EMT pathway, although it does not directly regulate *PRDM16* expression. Importantly, decitabine treatment can counteract the tumor-promoting effects induced by *PPARG* downregulation. Overall, these findings indicate that PRDM16 acts as a tumor suppressor in CRC through a DNA methylation-dependent mechanism involving the PPARG/EMT signaling pathway [36].

Given its involvement in EMT modulation and metastatic progression, *PRDM16*-related alterations are functionally consistent with CMS4 tumors. This subtype is characterized by mesenchymal activation, high stromal infiltration, and TGFβ-driven transcriptional programs, all of which overlap with *PRDM16*-mediated regulation of EMT and metastatic dissemination [8]. However, further studies are required to validate this association linking PRDM16 to CMS4 tumors. Interestingly, although observed in other cancer types, PRDM16 inhibits TGFβ signalling by stabilizing the inactive SMAD3-SKI complex on the promoter of TGFβ target genes [83,84].

An additional layer of complexity may derive from the context-dependent functions of PRDM16 observed in other tissues, where it regulates metabolic and differentiation programs. In CRC, it remains unclear whether *PRDM16* loss is purely tumor-epithelial or reflects broader ecosystem remodeling, including adipose–tumor interactions in visceral fat–rich microenvironments [16].

Moreover, as several PRDM family members exert isoform-dependent functions, systematic assessment of *PRDM16* transcript variants in CRC could clarify whether distinct isoforms contribute differently to EMT regulation and metastatic dissemination. Integrating isoform-resolved transcriptomics with CMS stratification may therefore refine its classification as a context-dependent tumor suppressor [18].

However, according to the dual role often observed for PRDM, *PRDM16-DT* (PRDM16 divergent transcript, also known as LINC00982) has been identified as a suppressor of metastasis and chemoresistance in CRC. Reduced expression of *PRDM16-DT* is significantly associated with aggressive tumor characteristics and poor clinical outcomes in CRC patients. Mechanistically, *PRDM16-DT* is transcriptionally regulated by FOXP3. It directly binds to HNRNPA2B1 and competitively inhibits its interaction with exon 9 of *CHEK2*. This interaction promotes the production of the long isoform of *CHEK2* (*L-CHEK2*), which enhances E-cadherin levels. Increased E-cadherin suppresses fibroblast activation, leading to reduced MMP9 secretion and inhibition of CRC metastasis. Furthermore, Cimicifugoside H-1, a natural bioactive compound, has been shown to bind specific residues of FOXP3 (LEU89, HIS91, and LEU92), thereby upregulating *PRDM16-DT* expression. This upregulation suppresses metastasis and improves sensitivity to oxaliplatin in CRC [37].

### 2.10. ZFPM2

*ZFPM2-AS1* is discussed here due to its functional convergence with PRDM-regulated pathways, although it does not belong directly to the PRDM protein family. Unlike *PRDMs*, *ZFPM2-AS1* is a long non-coding RNA and lacks a PR domain and zinc finger motifs. To date ZFPM2 has not been directly associated with CRC yet. Nevertheless, *ZFPM2-AS1* has been found to be significantly upregulated in CRC tissues and cell lines compared with normal colon mucosa and nonmalignant epithelial cells. Elevated *ZFPM2-AS1* expression correlates with aggressive clinicopathological features, including increased tumor size, poor histological differentiation, lymph node metastasis, and advanced TNM stage, suggesting a role in CRC progression [38]. Functional studies demonstrate that *ZFPM2-AS1* acts as an oncogene in CRC. *ZFPM2-AS1* silencing markedly suppresses cell proliferation, colony formation, migration, and invasion in vitro. Mechanistically, *ZFPM2-AS1* functions as a competing endogenous RNA (ceRNA), sponging miR-137 and thereby relieving post-transcriptional repression of the oncogenic factor TRIM24. Bioinformatic analyses and correlation studies reveal an inverse relationship between miR-137 and *TRIM24* expression, alongside a positive correlation between *ZFPM2-AS1* and *TRIM24*. Notably, *TRIM24* overexpression partially rescues the inhibitory effects of *ZFPM2-AS1* knockdown, confirming the functional relevance of this ceRNA regulatory axis [38,39]. Differential expression analyses between CRC and normal tissues, combined with integration of DElncRNAs, DEmiRNAs, and DEmRNAs, enabled construction of a CRC-specific ceRNA network. Within this network, *ZFPM2-AS1* emerges as a key prognostic biomarker, linking aberrant lncRNA expression to dysregulated miRNA–mRNA interactions that drive tumor progression. These findings provide a deeper understanding of prognosis-related ceRNA regulatory mechanisms in CRC and underscore the potential of *ZFPM2-AS1* as both a biomarker and therapeutic target [85].

Of note, further mechanisms involving *ZFPM2* could also occur. Indeed, in a different cancer type like lung adenocarcinoma, ZFPM2-AS1 promotes cancer progression by interacting with UPF1 to destabilize *ZFPM2* through mRNA decay [86]. However, current research is limited, and further investigation is needed to establish the direct involvement of *ZFPM2*.

## 3. Discussion

The PRDM family represents a functionally diverse group of epigenetic regulators whose deregulation contributes to multiple layers of CRC biology, including chromatin remodeling, transcriptional control, stemness, immune modulation, and therapeutic resistance. A unifying feature is the context-dependent and often isoform-specific behavior of PRDM proteins, which may act either as tumor suppressors or oncogenic drivers depending on genetic background, epigenetic landscape, and microenvironmental cues.

A central paradigm in PRDM biology is the “PR^+^/PR^−^ Yin–Yang” model, whereby full-length PR domain-containing isoforms generally exert growth-restrictive functions, whereas PR-deficient variants promote proliferation and survival [1,4]. This behavior is exemplified by PRDM2 in CRC. *PRDM2* is frequently disrupted in MSI tumors through frameshift mutations in coding mononucleotide tracts, leading to loss of the PR^+^ *PRDM2/RIZ1* isoform and relative predominance of the PR^−^ *PRDM2/RIZ2* variant [55,59]. Functional studies demonstrated that PRDM2/RIZ1 restoration induces G2/M arrest, apoptosis, and suppression of tumorigenicity [22,61], whereas PRDM2/RIZ2 promotes oncogenic transcriptional reprogramming, notably via EGF signaling activation and enhancement of proliferative and migratory capacity [14]. Epigenetic silencing of *PRDM2* in serrated lesions [13] further supports that *PRDM2* deregulation is an early and subtype-specific event in CRC evolution. Thus, isoform imbalance within PRDM2 provides a mechanistic link between epigenetic dysregulation, growth factor signaling, and tumor progression.

A similar tumor-suppressive role is observed for *PRDM5*, which is frequently silenced in CRC through promoter CpG island hypermethylation and H3K27 trimethylation [15,27]. Loss of *PRDM5* enhances WNT signaling and proliferative capacity, while its restoration induces cell-cycle arrest and apoptosis [26,76]. The enrichment of *PRDM5* methylation in CIMP-positive and *BRAF*-mutant tumors underscores its integration within epigenetically driven CRC subtypes and highlights the relevance of chromatin-based mechanisms in early tumorigenesis. Similarly, PRDM16 has been reported to exert tumor-suppressive effects through EMT modulation and interaction with PPARG, with DNA methylation–dependent silencing correlating with metastasis and poor prognosis [36]. Together, these findings reinforce the concept that epigenetic inactivation of PRDM tumor suppressors contributes to molecular plasticity and malignant progression.

In contrast, other PRDM members exhibit predominantly oncogenic behavior in CRC. *PRDM14* is highly expressed at the invasive tumor front and functions as an independent prognostic factor in advanced disease [31,79]. Mechanistically, PRDM14 intersects with Hippo–YAP1 signaling and can compensate for YAP1 loss, sustaining tumor growth through transcriptional activation of CALM2 and SLC2A1 [32]. PRDM15 similarly promotes CRC progression by repressing *USP10* and destabilizing TP53, thereby attenuating TP53-dependent cell-cycle arrest and apoptosis [35]. Moreover, PRDM15 participates directly in DNA damage repair through recruitment to double-strand breaks and engagement of the DNA-PK complex, contributing to radio resistance [34]. These data suggest that certain PRDMs act as regulators of genome stability and therapy response, positioning them as potential determinants of treatment failure.

MECOM further illustrates the complexity of PRDM family functions. While early studies identified *MECOM* as a target of frameshift mutation in MSI-high CRC [65], accumulating evidence supports a predominantly oncogenic role in advanced disease. *MECOM* overexpression correlates with poor differentiation, metastasis, and adverse outcomes [64]. Mechanistically, EVI1 promotes metastatic competence through epigenetic repression of metastasis suppressor genes such as *TIMP2* via DNMT1 recruitment [23], modulates TGFβ signaling [69], regulates KRAS signaling via miR-143 repression [68], and influences glycosylation programs associated with metastatic potential [72,73]. However, context-dependent tumor-suppressive functions have also been described in inflammation-driven models [71], reinforcing the notion that PRDM activity is shaped by microenvironmental and genetic context.

PRDM1 (BLIMP1) provides an additional example of functional duality. In CRC cells, PRDM1 represses MYC-driven transcriptional programs and stemness-associated genes, partly within TP53-dependent networks [20,42]. Pharmacologic induction of *PRDM1* results in cell-cycle arrest through MYC repression and CDKN1A upregulation, supporting a tumor-suppressive role. However, stress-induced ribosomal dysfunction elevates *PRDM1* expression and promotes tumor progenitor survival and stemness through IGF-related signaling pathways [32]. Moreover, PRDM1 contributes to immune regulation, including T-cell exhaustion phenotypes [21], highlighting its capacity to shape both tumor-intrinsic and microenvironmental programs. This context dependency likely reflects differences in isoform expression, chromatin occupancy, and interaction partners across CMSs.

Collectively, current evidence indicates that PRDM family proteins function as key integrators of signaling heterogeneity across the CMS of CRC. Rather than acting independently, PRDM members are embedded within subtype-specific regulatory networks that shape tumor behavior, plasticity, and therapeutic response.

Distinct PRDM proteins are associated with specific molecular contexts. Alterations in *PRDM2* and *PRDM5* are frequently enriched in MSI and CIMP backgrounds. PRDM14 is linked to proliferative and stemness-associated pathways characteristic of CMS2 and CMS4 tumors, while PRDM15 correlates more strongly with the aggressive and therapy-resistant characteristics of CMS4. MECOM contributes to EMT, TGF-β signaling, and inflammatory pathways, whereas PRDM1 influences TP53-mediated stress responses as well as modulation of the tumor immune microenvironment. Together, these findings support a model in which PRDM proteins coordinate diverse oncogenic programs in a subtype-dependent manner.

From a translational standpoint, PRDM alterations represent promising candidates for biomarker development and therapeutic targeting. For example, the epigenetic silencing of *PRDM5* and *PRDM2* suggests potential sensitivity to demethylating agents, which could be exploited in biomarker-guided therapeutic strategies. In this context, stratifying patients based on *PRDM2* isoform imbalance (RIZ1/RIZ2 ratio) or *PRDM5* promoter methylation status may guide the selection of epigenetic therapies in CRC, as these alterations have been linked to tumor progression and early tumorigenesis [13,14,15].

Moreover, given the role of PRDM15 in DNA damage response and radiotherapy resistance, its inhibition may represent a rational strategy to enhance radiotherapy efficacy in resistant rectal cancer, particularly in combination with standard chemoradiotherapy regimens [34,35]. Such integrative analyses may uncover context-specific dependencies and therapeutic vulnerabilities.

Future studies should specifically address the isoform-resolved functions of PRDM proteins across CRC molecular subtypes, including CMS-stratified cohorts, to better define their context-dependent roles.

In addition, integrating spatial transcriptomics and single-cell approaches will be essential to distinguish tumor-intrinsic versus microenvironmental contributions of PRDM signaling [16,18].

Finally, preclinical studies evaluating PRDM-targeted interventions, including isoform-specific modulation and combination strategies with standard therapies (e.g., EGFR-targeted agents or radiotherapy), will be crucial to translate current knowledge into clinically actionable approaches [14,34].

Consistent with the strategies outlined above, PRDM15 and MECOM have been implicated in resistance to chemotherapy and radiotherapy, highlighting opportunities for rational combination therapies. In addition, *PRDM14* methylation detected in circulating DNA and *PRDM1* expression in immune cells may serve as minimally invasive biomarkers, detectable in peripheral blood, for disease monitoring. Nevertheless, therapeutic strategies must carefully consider isoform specificity and context-dependent effects to avoid unintended activation of pro-survival signaling pathways.

Despite substantial advances in understanding the role of PRDM proteins in cancer, several critical questions remain unresolved. The consequences of disrupting the balance among PRDM isoforms require further investigation, as isoform-specific functions may differentially influence tumor progression. Moreover, the broader regulatory architecture of PRDM proteins—including their upstream regulators, downstream effectors, and protein interaction networks—has not yet been systematically characterized. This knowledge gap limits the rational design of targeted interventions.

Comprehensive multi-omics approaches integrating transcriptomic, proteomic, epigenomic, and metabolomic data will be essential to elucidate the dynamic regulatory landscapes governed by PRDM proteins.

Transcriptomic and proteomic analysis performed on CRC cells and *PRDM* overexpressing or silenced cells may be analyzed to discover overexpressed RNAs and proteins that could be validated in serum CRC patients as possible circulating biomarkers with the potential pathological signature for CRC diagnosis and/or monitoring.

In the same experimental setting, methylome analysis could reveal the target sites of PRDMs in the genome.

To assess the role of PRDMs on the bioenergetic and biosynthetic pathways, CRC cells, and the *PRDM* overexpressing or silenced cells may be analyzed through untargeted NMR-based metabolomics approach. Findings will be validated by target metabolomics analyses that will rely upon NMR, LC-MS and GC-MS approaches (depending on the target metabolites).

Several cell partners and molecular interactions of PRDMs remain poorly characterized. Identifying these interactions could provide pivotal insights into how *PRDM* dysregulation contributes to CRC. Comparative analysis performed on overexpressing and or silenced *PRDM* cells will be useful for this purpose. Advances in mass spectrometry technologies, together with improved computational approaches, now enable the high-confidence mapping of protein interaction networks, thereby enhancing our understanding of protein functions and their biological roles. In this framework, comprehensive transcriptome profiling using next-generation sequencing, combined with proteomic analyses, could help identify the full set of PRDM target genes and delineate the pathways underlying their role in CRC. Accordingly, integrating transcriptomic data with the characterization of the PRDM interactome represents a key step toward clarifying the molecular mechanisms that drive PRDM function.

Furthermore, the development of isoform-selective small-molecule agonists or inhibitors represents a promising avenue. For instance, the ARIZ-047 siRNA which specifically targets the oncogenic RIZ2 isoform can rebalance the expression ratio toward the RIZ1 tumor suppressor, displaying significant antitumor effects in lung cancer, both in vitro and in vivo, through WNT pathway modulation [87]. Such an approach represents a promising strategy for precision medicine applications also in CRC.

Additionally, a compelling yet challenging strategy involves targeting the HMT activity of PRDMs. To date, it has been discovered the MRK-740, a selective and potent PRDM9 inhibitor, that directly binds the substrate-binding pocket [88].

Advancing the clinical translation of PRDM research—including validation of diagnostic and prognostic biomarkers and the design of personalized therapeutic regimens based on PRDM-associated pathways—could significantly enhance their impact within precision oncology.

## 4. Conclusions

The PRDM family represents a network of epigenetic regulators that collectively shape CRC initiation, progression, subtype heterogeneity, and therapeutic response. Their dual enzymatic and scaffold functions, combined with isoform diversity and context-dependent activity, position PRDM proteins as key modulators of chromatin plasticity in CRC carcinogenesis. Future studies integrating isoform-resolved transcriptomics, chromatin occupancy profiling, and CMS-stratified functional studies will be essential to fully elucidate their biological and clinical significance.

## Figures and Tables

**Table 1:** Overview of PRDM family members in CRC, including molecular alterations, functional roles, key mechanistic pathways, CMS associations, and clinical relevance.

Gene Symbol	Main Alteration	Functional Role	Key Mechanistic Axis	CMS Context	Clinical Relevance	References
*PRDM1* *(BLIMP1)*	Downregulation; stress-induced upregulation; immune-compartment expression	Context-dependent (tumor suppressor/immune regulator)	MYC repression; IGF signaling; T-cell exhaustion	CMS1 (direct immune association)	Prognostic (immune context); potential immunomodulatory target	[19,20,21]
*PRDM2* *(RIZ)*	MSI frameshift mutations; promoter hypermethylation (RIZ1); isoform imbalance (RIZ2 overexpression)	Dual (RIZ1 tumor suppressor/RIZ2 oncogenic)	H3K9 methylation; EMT regulation; EGF/EGFR signaling	MSI-associated (likely CMS1); CMS2 alignment (EGFR axis)	Predictive (EGFR response?); prognostic; early serrated lesion marker	[13,14,22]
*MECOM* *(PRDM3/MDS1/EVI1)*	Overexpression; enhancer-mediated activation; MSI-associated frameshift	Predominantly oncogenic (context-dependent)	TGFβ disruption; TIMP2 repression; KRAS/miR-143; glycosylation programs	Conceptual alignment with CMS4	Prognostic; predictive (CRT models); therapeutic target candidate	[23,24,25]
*PRDM5*	Promoter CpG hypermethylation; H3K27me3 enrichment	Tumor suppressor	Wnt pathway repression; serrated pathway epigenetic silencing	CIMP/MSI-associated (CMS1 enrichment)	Early epigenetic biomarker; potential prognostic marker	[15,26,27]
*PRDM10*	Differential expression (rectal cancer grade II vs. III); mutation/overexpression in TGCA datasets	Putative transcriptional regulator of tumor survival	BCL2 transcriptional regulation; apoptosis modulation	Not CMS-stratified	Possible prognostic relevance (grade-associated); functional role requires validation	[10,28,29]
*PRDM12*	Promoter methylation in CIMP tumors	Epigenetic biomarker (functional role unclear)	Multi-marker methylation panel	Hypothesized CMS1 (CIMP-associated)	Early detection biomarker (stool DNA panels)	[30]
*PRDM14*	Overexpression; cfDNA hypermethylation	Oncogene	YAP1/Hippo compensation; CALM2/SLC2A1 activation	Conceptual alignment with CMS4	Prognostic marker; blood-based biomarker candidate	[31,32,33]
*PRDM15*	Overexpression; DNA damage–induced activation	Oncogene	USP10–TP53 repression; DNA-PK/DSB repair	Hypothesized CMS4 (therapy-resistant niche)	Predictive (radiotherapy response); therapeutic vulnerability	[34,35]
*PRDM16*	Reduced expression; DNA methylation; PRDM16-DT dysregulation	Predominantly tumor suppressor (context-dependent)	PPARγ/EMT axis; isoform diversity	Conceptual alignment with CMS4	Prognostic marker; metastasis regulator; therapy-sensitization potential	[36,37]
*ZFPM2-AS1*	Overexpression (lncRNA)	Oncogene	ceRNA (miR-137/TRIM24 axis)	Not CMS-stratified	Prognostic biomarker; therapeutic target candidate	[38,39]

Abbreviations: CRC, colorectal cancer; CMS, consensus molecular subtype; MSI, microsatellite instability; CIMP, CpG island methylator phenotype; CRT, chemoradiotherapy; EMT, epithelial–mesenchymal transition; EGFR, epidermal growth factor receptor. CMS context classification: “Direct Association” indicates studies explicitly analyzing PRDM expression or function in CMS-stratified CRC cohorts. “MSI-associated” refers to alterations enriched in MSI-high tumors, which frequently correspond to CMS1. “Conceptual alignment” denotes mechanistic overlap with signaling programs characteristic of a given CMSs, without direct CMS-stratified validation. “Hypothesized” indicates biologically plausible subtype association based on pathway involvement, pending formal CMS-based investigation.

## Data Availability

No new data were created or analyzed in this study. Data sharing is not applicable to this article.

## Abbreviations

The following abbreviations are used in this manuscript:

ApcMin	Adenomatous polyposis coli Multiple intestinal neoplasia
BCL-xL	B-cell lymphoma-extra large
CALM2	Calmodulin 2
CHEK2	Checkpoint Kinase 2
CIMP	CpG Island Methylator Phenotype
CMS	Consensus Molecular Subtype
CRC	Colorectal Cancer
CSC	Cancer Stem Cell
DNA-PKcs	DNA-dependent Protein Kinase catalytic subunit
DNMT1	DNA Methyltransferase 1
DSB	Double-strand Break
EGF	Epidermal Growth Factor
EGFR	Epidermal Growth Factor Receptor
EMT	Epithelial–Mesenchymal Transition
ETS2	ETS proto-oncogene 2
FOXP3	Forkhead box P3
FUT3	Fucosyltransferase 3
GEO	Gene Expression Omnibus
H3K27me3	Trimethylation of histone H3 at lysine 27
H3K9me2	Dimethylation of histone H3 at lysine 9
HNRNPA2B1	Heterogeneous Nuclear Ribonucleoprotein A2/B1
IBD	Inflammatory Bowel Disease
IGF	Insulin-like Growth Factor
IGF-1R	Insulin-like Growth Factor 1 Receptor
lncRNA	Long non-coding RNA
MAPK	Mitogen-activated Protein Kinase
MECOM	MDS1 and EVI1 Complex locus
MeDIP-seq	Methylated DNA Immunoprecipitation sequencing
miRNA	MicroRNA
MMP9	Matrix Metalloproteinase 9
MSI	Microsatellite Instability
MSI-H	Microsatellite Instability–high
MSS	Microsatellite Stable
PKD1	Protein Kinase D1
PPARG	Peroxisome Proliferator-activated Receptor Gamma
PRDM	PR omain-containing protein
PR^+^	PR domain-positive isoform
PR^−^	PR domain-negative isoform
RCC	Right-sided Colorectal Cancer
RIZ	Retinoblastoma-interacting Zinc finger
sDNA	Stool DNA
SE	Super-Enhancer
SET	Su(var)3-9, enhancer-of-zeste, trithorax
TGFβ	Transforming Growth Factor beta
TIMP2	Tissue Inhibitor of Metalloproteinases 2
TNM	Tumor–Node–Metastasis
TP53	Tumor Protein p53
TRIM24	Tripartite Motif containing 24
USP10	Ubiquitin-Specific Peptidase 10
VAT	Visceral Adipose Tissue
VEGF	Vascular Endothelial Growth Factor
VEGFR	Vascular Endothelial Growth Factor Receptor
VIM	Vimentin
WNT	Wingless-related integration site
YAP1	Yes-associated Protein 1
ZFPM2-AS1	ZFPM2 Antisense RNA 1.
